# Investigating Hybridization between the Two Sibling Bat Species *Myotis myotis* and *M*. *blythii* from Guano in a Natural Mixed Maternity Colony

**DOI:** 10.1371/journal.pone.0170534

**Published:** 2017-02-15

**Authors:** Eve Afonso, Anne-Claude Goydadin, Patrick Giraudoux, Gilles Farny

**Affiliations:** 1 Laboratoire Chrono-environnement UMR CNRS 6249, Université de Bourgogne Franche-Comté, Besançon, France; 2 Institut Universitaire de France, Paris, France; 3 Parc National des Ecrins, Domaine de Charance, Gap, France; Università degli Studi di Napoli Federico II, ITALY

## Abstract

Because they can form seasonal mixed-species groups during mating and maternal care, bats are exciting models for studying interspecific hybridization. *Myotis myotis* and *M*. *blythii* are genetically close and morphologically almost identical, but they differ in some aspects of their ecology and life-history traits. When they occur in sympatry, they often form large mixed maternity colonies, in which their relative abundance can vary across time due to a shift in the timing of parturition. For the first time, we used non-invasive genetic methods to assess the hybridization rate and colony composition in a maternity colony of *M*. *myotis* and *M*. *blythii* located in the French Alps. Bat guano was collected on five sampling dates spread across the roost occupancy period and was analysed for individual genotype. We investigated whether the presence of hybrids followed the pattern of one of the parental species or if it was intermediate. We identified 140 *M*. *myotis*, 12 *M*. *blythii* and 13 hybrids among 250 samples. Parental species appeared as genetically well-differentiated clusters, with an asymmetrical introgression towards *M*. *blythii*. By studying colony parameters (effective size, sex ratio and proportion of the three bat types) across the sampling dates, we found that the abundances of hybrid and *M*. *blythii* individuals were positively correlated. Our study provides a promising non-invasive method to study hybridization in bats and raises questions about the taxonomic status of the two *Myotis* species. We discuss the contribution of this study to the knowledge of hybrid ecology, and we make recommendations for possible future research to better understand the ecology and behaviour of hybrid individuals.

## Introduction

Natural hybridization is the successful mating of genetically distinguishable groups or taxa that leads to the production of viable hybrids [1,2]. Once considered a rare phenomenon in animal species, natural interspecific hybridization may be relatively common: 10% of animal species may be involved in hybridization and potential introgression with other species [2]. Although the role of hybridization has been debated, hybrids may contribute to the adaptive variation of existing species through the spread of favourable alleles [2–4].

Even though bats currently include over 20% of mammal species around the world [5], many aspects of their ecology are still unknown, especially regarding interspecific hybridization. Several studies have demonstrated the occurrence of past or actual hybridization or introgression using molecular methods, but these events involve fewer than 20 (1.6%) of the 1,260 bat species known worldwide [5–11]. This may be associated more with the lack of studies focusing on bat species than with the higher reproductive isolation between bat species compared to other mammals. However, the life-cycle of bats makes bat species fascinating models for studying hybridization, as they can form seasonal mixed-species groups during mating [12] and/or maternal care [13]. The few reports of the frequency of hybrid individuals suggest that interspecific hybridization could be substantial in bat species, as it may represent 1.7% to 14% of the sampled individuals, depending on the species studied (Table 1). These estimations are comparable to those reported for other mammalian species [14, 15]. In the vast majority of cases, these results are only based on few samples collected over extensive large geographical areas and are difficult to extrapolate to natural populations or seasonal colonies. Thus, such an occurrence of hybridization events raises questions about the ecology of bat populations, particularly in the case of cryptic species living in sympatry.

**Table 1:** Reports of interspecific hybridization between bat species.

Hybridizing species			Frequency of hybrid individuals	Nature and location of the sample	Molecular methods	References
*Miniopterus schreibersii schreibersii*	*Miniopterus schreibersii pallidus*	-	14% (3/21)	Large-scale sample (Turkey)	mtDNA and ncDNA markers	[16]
*Pipistrellus pipistrellus*	*Pipistrellus pygmaeus*	-	1.7–13.3% (3/176–23/173)	Large-scale sample (Poland)	Microsatellite markers	[17]
*Myotis alcathoe*	*Myotis brandtii*	*Myotis mystacinus*	3.2–7.2%(12/375–16/222)	Large-scale sample (Poland)	Microsatellite markers	[12]
*Myotis myotis*	*Myotis blythii*	-	10%(16/160)	Large-scale sample (Europe)	mtDNA and Microsatellite markers	[7]

The two sibling bat species *Myotis myotis* (the greater mouse-eared bat) and *M*. *blythii* (the lesser mouse-eared bat) are morphologically almost identical [18,19]. The small genetic distance between the two species and reported cases of hybridization suggest incomplete reproductive isolation [7, 9, 19]. Despite their morphological and genetic proximity, the two bat species differ in their ecology and in some life-history traits. The diet of *M*. *myotis* includes mainly carabid beetles, whereas bush crickets constitute the greatest proportion of the diet of *M*. *blythii* [20, 21]. This dietary difference prompts both a difference in foraging habitats and a shift in the timing of parturition. The primary foraging habitat of the two bat species corresponds to the habitat requirement of their main prey: habitats selected by *M*. *myotis* are characterized by high accessibility to ground-dwelling prey (freshly cut meadows, forest without underground), while grasslands predominate in all *M*. *blythii* habitats [22]. Additionally, the timing of parturition of the two bat species correlates with the availability of their respective main prey; consequently, *M*. *blythii* can arrive to the roost later than *M*. *myotis* and gives birth 10 days after *M*. *myotis* [13]. However, the two *Myotis* species frequently occupy the same maternity roosts and form mixed roosting groups containing from 50 to 1000 females and young [23]. Hybrids are examined mainly for genotypic and/or phenotypic variations compared to the parental species in the study of wildlife species in general [2, 4]; thus, nothing is known about species assemblage in such bat guilds. These mixed-species groups therefore provide exciting perspectives to study hybrids, especially roost time sharing relative to parental species.

Because these two species are not readily distinguishable morphologically, correct species determination during visual counts of individuals in large mixed colonies is not possible. A combination of several morphological and morphometric measurements based on dimensions of the skull, tooth row construction or external features (white spot, ear size, forearm length) can help to distinguish the two species and potentially hybrid individuals [18, 19, 24]. However, individuals are sometimes technically and ethically inaccessible with respect to capture and handling. Furthermore, invasive methods, such as using a sterile biopsy punch of the wing membrane, limit sample size for the same reasons. Alternative non-invasive and non-disruptive methods based on the collection of non-invasive samples (*i*.*e*., hair and faeces) are increasingly used to study population genetics and have been used with success in studying bat species [25]. In the present study, we sampled a mixed maternity colony of *M*. *myotis* and *M*. *blythii* located in the French Alps through sampling and analysis of their guano, taking advantage of recent developments in non-invasive approaches in wildlife studies. As hybrids were detected in two mixed-species maternity colonies located in the Alps (Valais, Switzerland) and their southern border (Aglié, Italy), with 10% of the sampled individuals presenting admixed genotypes [7], we expected to detect hybridization cases. Bats were sampled at different times throughout the occupancy period of the roost. We investigated whether non-invasive methods permit the detection of hybrid individuals and, if so, whether the hybrids were present at different times during the roost occupancy period, following the pattern of one of the parental species or at an intermediate time. Non-invasive methods were also used to assess colony parameters (colony size and sex ratio). This article illustrates how non-invasive methods can be used to estimate demographic parameters of species difficult to access directly.

## Materials and Methods

### Dropping collection

The study took place in the attic space of a former children’s day nursery in Le Bourg d’Oisans (45.06° N, 6.03° E) in the French Alps. This bat maternity roost includes several hundred individuals of both *M*. *myotis* and *M*. *blythii* and is frequented from April to October every year. The mixed bat colony is composed of females that gather to give birth and raise their young.

Samples were collected every 3–4 weeks by the officers of The Ecrins National Park, from May to August 2012 when individuals were abundant in the roost. We expected to sample mainly adult females in May because no young had been born during that month. Births generally occur in June in the study site (first births in early June, Gilles Farny, comm. pers.). One can therefore assume that droppings from young could have been sampled from July to August, when young individuals reach adult body size. Plastic sheets were placed on the ground beneath the areas occupied by bats and were left for 24 to 48 hours. Droppings were then randomly collected directly from the plastic sheets, placed individually in 2-mL microtubes and frozen (-20°C) until analysis. A total of 250 droppings were collected on 5 dates (50 droppings per sampling date) spread across the study period (Table 2).

**Table 2:** Number of samples successfully genotyped and number of individuals sampled by sampling date.

Sampling date (2012)	Number of dropping samples	Number of samples successfully genotyped	Number of individuals	Average visual count	Eggert estimation of colony size	Bayesian estimation of colony size	Overall sex ratio
May 22	50	43	41	400	433 [426; 441]	1414 [138; 3901]	0.03
June 20	50	36	31	360	117 [115; 118]	194 [56; 416]	0.04
July 10	50	45	43	230	465 [458; 472]	1486 [153; 3981]	0.05
August 03	50	44	41	240	307 [301; 314]	779 [102; 2231]	0.03
August 29	50	42	28	40	46 [45; 47]	56 [34; 81]	0.27
Total	250	210	165	-	-	-	-

The overall sex ratio is the number of males divided by the total number of sexed individuals.

### Ethics statement

Field sampling was carried out in a communal building with the authorisation of the mayor of Bourg d’Oisans. Samples collection and transport were permitted from the Authority of the Ecrins National Park (permit number 222/2012). This study being based on droppings, no animal was captured or disturbed during the study period. No other permits were required.

### DNA extraction and microsatellite genotyping

DNA was extracted from the droppings using the QIAamp DNA Stool Mini Kit (Qiagen, Courtaboeuf, France) following the protocol detailed in [26]. Each sample was processed independently in an automated manner using the QIAcube robot (Qiagen). DNA extracts were stored at -20°C until DNA amplification.

Genotyping was carried out by amplifying 12 microsatellite markers: 3 markers (C113, D15, F19) developed by Castella and Ruedi [27] and 9 markers (B8-Mluc, D15-Mluc, EF15-Mluc, F19-Mluc, G2-Mluc, G6-Mluc, G30-Mluc, G31-Mluc, H23-Mluc) developed by Jan et al. [28]. DNA amplification was performed in two different multiplex reactions (Table 3). Each reaction was performed in a mixture (14 μL) consisting of 4 μL of DNA extract, 1× Multiplex PCR Master Mix (Qiagen), and primer concentrations as reported in Table 3. The amplification programme comprised an activation step of 15 min at 95°C, followed by 35 cycles of denaturation at 94°C for 30 s, annealing at 57°C for 90 s, and primer extension at 72°C for 60 s. A final extension was performed for 30 min at 60°C. Amplification reactions were performed using an Eppendorf Mastercycler DNA Engine. PCR products were diluted in 24 μL of ultra-pure water and stored at -20°C. Allele size was quantified using an Applied Biosystems 3130 Genetic Analyzer in a reaction containing 1 μL of the diluted PCR product, 0.25 μL of an internal lane standard (500-LIZ; Applied Biosystems) and 9.75 μL of deionized formamide. Genetic profiles were acquired using the program Genemapper version 3.7 (Applied Biosystems).

**Table 3:** The 12 microsatellite markers used in the present study.

Microsatellite marker	Forward (F) and reverse (R) primer sequence (5’-3’)	Primer concentration (μM)	Allele frequency differential (δ)
**Panel 1**			
D15	F: [VIC] 5’–GCTCTCTGAAGAGGCCCTG– 3’ R: 5’–ATTCCAAGAGTGACAGCATCC– 3’	0.200.20	0.62
EF15-Mluc	F: [PET] 5’–GATCGCAGTCCCTTCC– 3’ R: 5’–GCTTATGGGGAGAAATGAG– 3’	0.270.27	-
F19	F: [VIC] 5’–GCTAGCCATGGAGAAGGAAG– 3’ R: 5’–CCCAAATCTGTCTTTCAGGC– 3’	0.200.20	0.50
G2-Mluc	F: [FAM] 5’–TGAAAAGAACTGGAGAGGCTTT– 3’ R: 5’–AGATTGATGAATGTGAAAGGTCAG– 3’	0.200.20	0.71
G6-Mluc	F: [PET] 5’–GGCTTTTTGAAAAGACTGAGG– 3’ R: 5’–ACATCAGCCAGTTCCTGTTC– 3’	0.200.20	0.25
G31-Mluc	F: [FAM] 5’–GATCACCAATCATGTAAGGTTCAC– 3’ R: 5’–AAGTCAAGGCCAAGCAAGTC– 3’	0.200.20	0.68
**Panel 2**			
B8-Mluc	F: [VIC] 5’–AAATACCTGAGTGAGAACATTTAGTGGAG– 3’ R: 5’–CTCATTAACTTCATTGGTAAGTGTTGTACC– 3’	0.400.40	0.50
C113	F: [FAM] 5’- ACCTCCCTGCCCTGCAC– 3’ R: 5’–GCAATGCTTCCTCCAAGTCC– 3’	0.200.20	0.79
D15-Mluc	F: [NED] 5’–AAATTCTTTCCCTCCAAAGTGG– 3’ R: 5’–GCACGCTCAGACTCCTTCC– 3’	0.200.20	0.66
F19-Mluc	F: [PET] 5’–TGTAGCTAGCCATGGAGAAGG– 3’ R: 5’–AAATGGTTACATTACAGAAAATGCTC– 3’	0.200.20	0.59
G30-Mluc	F: [PET] 5’–GGCATGAACATGGAGTGAGG– 3’ R: 5’–GCTAGAAGTTATGGTCAATGTTCCTG– 3’	0.200.20	0.71
H23-Mluc	F: [VIC] 5’–TTGTCTACTAGCATTTGTCCAGTG– 3’ R: 5’–ATAGCTATGTTGCCTAACCTATTTACTC– 3’	0.400.40	0.43

The table provides the primers used in a specific multiplex PCR. The letters in square brackets indicate the fluoro-dyes used in one of the two sequences of each primer pair.

Multilocus genotypes were determined using a comparative multiple-tubes approach to reduce genotyping errors [29]. Two PCRs were performed for each sample and each multiplex reaction. A consensus genotype was defined for each locus; an allele was accepted only if it was recorded twice. Samples exhibiting inconsistencies between replicates or samples for which consensus genotypes were not complete (*i*.*e*., comprised fewer than 12 loci) were discarded from the analysis. PCR negative controls (including ultra-pure water instead of DNA extract) were regularly used to ensure the absence of cross contamination. Two samples were considered to originate from a single individual when the two multilocus genotypes were identical or when they differed by only one allele. Multiple comparisons between genetic profiles were performed using the allelematch package [30] in the R 3.1.0 software (R Development Core Team. R: A Language and Environment for Statistical Computing, R Foundation for Statistical Computing, Vienna, Austria, 2011. http://www.R-project.org).

### Hybrid detection and assignment to a genetic group

Multilocus genotypes were analysed using a Bayesian clustering method implemented in the NEWHYBRIDS software (version 1.1 beta; [31]). This method assigns individual multilocus genotypes to genetic clusters based on a Markov chain Monte Carlo (MCMC) simulation procedure to estimate the posterior distribution reflecting the membership of each individual.

The sample is taken from a mixture of pure individuals and hybrids [31]. All the individuals were genotyped with the same set of microsatellites, amplifying both *M*. *myotis* and *M*. *blythii* DNA. However, allele frequencies are known to vary between the two parental species, and their potential hybrids [7]. The programme estimates the allele frequencies in two putative parental populations determined by the software without prior information. The posterior probability of being of pure or hybrid origin is then estimated for each genotype. NEWHYBRIDS obtained the posterior distributions based on an MCMC procedure with a burn-in of 10^5^ steps, followed by a sampling period of 10^6^ steps. Under this model, the posterior probability *q* describes the probability that an individual belongs to each of the different genetic clusters. Two threshold values (*Tq* ≥ 0.75 or 0.90) were used with two different rules of assignment [32, 33]: (1) all individuals with a *q* ≥ *Tq* were considered purebred parentals, and all others were considered hybrids (no individual remained unassigned; 3^rd^ criterion); (2) all hybrid categories (F1, F2, backcrosses) were combined to identify admixed individuals without distinguishing hybrid categories (2^nd^ criterion); and individuals with a *q* < *Tq* for either purebred or hybrid categories were then unassigned. We omitted the most restrictive criterion (1^st^ criterion) in which the threshold value is applied to each category (purebreds, F1, F2, backcrosses) separately because only 12 markers were used in the study, which is too few to confidently assign all of the hybrid categories [12].

The possibility that the results obtained from the NEWHYBRIDS analyses could be observed by chance was tested by simulation studies following the protocol used by Burgarella et al. [33]. Simulated datasets were used to determine which method (*Tq* ≥ 0.75 or *Tq* ≥ 0.90; 2^nd^ or 3^rd^ criterion) provided the most reliable results to avoid the false assignment of individuals based on characteristics of the observed dataset [32]. Two subsamples, including individuals with the highest *q*-values (30 for *M*. *myotis* and 11 for *M*. *blythii*), were created. Datasets were simulated based on the allele frequencies calculated in the two subsamples with HYBRIDLAB 1.0 software: 10,000 genotypes were generated for both parental species, and 10,000 for each type of hybrid (F1, F2, and backcross). Genotypes were then randomly selected without replacement using the R 3.1.0 software to create a sample of 200 individuals with different proportions of hybrids (0%, 5%, 10%). For each hybrid proportion, 20 different simulated datasets were generated. The size of the simulated sample (200 individuals) and the hybrid proportions were chosen to represent the sample collected in this study. Each simulated sample was analysed with NEWHYBRIDS according to the same setting conditions, threshold values and criteria as those described above. The following measures were then calculated to evaluate the performance of the methods [32, 33]: (1) the hybrid proportion (HP) (*i*.*e*., the number of individuals classified as hybrids over the total number of individuals in the sample); (2) the efficiency in detecting the true hybrid/purebred status of individuals (*i*.*e*., the number of correctly identified individuals for a category over the actual number of individuals of that category in the sample); (3) the accuracy (*i*.*e*., the number of correctly identified individuals for a category over the total number of individuals assigned to that category); and (4) the type I error (*i*.*e*., the number of individuals wrongly identified as hybrids over the total number of actual purebreds in the sample).

Because we collected faeces instead of examining individuals, we used bat corpses to help to assign purebred clusters to the two *Myotis* species. Bat corpses were collected as soon as they were found within the roost on the different sampling dates. Morphometric and morphological criteria were used identify the species of each bat corpse [18, 19]. The multilocus genotypes of the four individuals collected (2 *M*. *myotis* and 2 *M*. *blythii*) were included in a clustering analysis to assign clusters to the parental species and excluded for the analysis of guano samples.

### Genetic differentiation between inferred groups of bats

Once all of the bats were assigned as *M*. *myotis*, *M*. *blythii* or hybrids, genetic diversity was assessed for the three inferred bat types. The allelic richness per bat type was estimated based on a rarefaction procedure implemented in the R package hierfstat [34]. Genotypic linkage disequilibria between all pairs of loci and conformation to the Hardy—Weinberg equilibrium (HWE) for each locus separately and over all loci were tested within each bat type by exact tests using Markov chain methods in GENEPOP software version 4.1.4 [35]. Corrections for multiple tests were performed using the false discovery rate (FDR) approach using the R software. The genetic differentiation between the three bat types was then quantified by computing the Weir and Cockerham [36] estimator of *F*_ST_ using GENEPOP.

### Estimation of colony size

Statistical approaches were used to evaluate whether colony size could be reliably estimated in such mixed-species groups. Bat colony size is sometimes difficult to readily assess by visual counting within maternity roosts. The number of bats in the roost varies daily, and individuals are difficult to count when they are located in inaccessible places or when they are flying within the roost. Estimators of population size can be used as an alternative to visual counting, but they have strong key assumptions linked to capture-mark-recapture methods, the most restrictive being demographic closure during the study period (*i*.*e*., no death, birth, emigration, immigration). Because our sampling method did not meet this assumption, we estimated colony size by two methods and compared the results to data from visual counting. Colony size was then estimated for each sampling date by 1) the sequential Bayesian estimator developed by Petit and Valière [37] and implemented in the R software with a script provided by Eric Petit (University Rennes 1, France), and 2) Eggert’s equation, a statistical model based on the concept of the rarefaction curve [38]. As the number of bats in the roost could vary widely each day, we reported the average number of bats usually seen in the roost at a given moment of the year rather than a punctual visual counting. These average numbers of individuals were assessed as the mean number of bats counted in the roost during the preceding three years during the same period of the year, with a period being defined as a 15-day stretch.

### Molecular sexing

To determine the sex ratio on each sampling date, the faeces were typed using sex-specific PCR [39]. Part of the *Sry* gene was amplified by duplex PCR using two sets of primers. A 447/445-bp region of the *Zfy-Zfx* genes was amplified as a positive control using XP15EZ/XP23EZ primers [40], and a 202-bp fragment of the SRY-HMG box of the *Sry* gene located on the Y chromosome was amplified for males using the SRYhmg-F/SRYhmg-R primers [41]. PCR amplifications were conducted in a reaction mixture (12 μL) consisting of 5 μL of DNA extract, 1× HotStarTaq Master Mix (Qiagen), and 0.3 μM of each primer and ultra-pure water. A negative control (ultra-pure water) was included for every 20 samples. The amplification cycling programme consisted of an activation step of 15 min at 95°C, followed by 32 cycles of denaturation at 93°C for 1 min, annealing at 50°C for 1 min, and primer extension at 72°C for 1 min. A final extension was performed at 72°C for 5 min. The PCR products were separated and visualized using the QIAxcel device (an automated capillary electrophoresis system produced by Qiagen) using the QIAxcel DNA high-resolution kit (Qiagen).

### Statistical analyses

We tested the correlation between the abundance of hybrid individuals and each of the two parental species using Pearson’s correlations. Linear models were checked graphically for homoscedasticity, and normality of models error was tested using a Shapiro-Wilk test.

## Results

### Sample composition and colony size

Among the 250 samples collected from May to August 2012, genotyping analyses gave consistent results for 210 samples (40 samples were excluded from the analysis because of incomplete genotyping results). The amplification success ranged from 72% (June 20) to 90% (July 10), which is consistent with previous studies based on bat guano [26, 29]. Eleven of the 12 microsatellites used for the genotyping analyses were polymorphic for all samples. The microsatellite EF15-Mluc showed only one allele (211) for all individuals. This marker was then discarded from the analyses; thus, 11 microsatellites were used to characterize the genetic profiles of all of the individuals. Our 210 genotyped samples were from 165 distinct individuals (1 to 4 samples collected per individual). For each of the five sampling dates, 28 to 43 distinct individuals were sampled (Table 2).

Individual gender was determined for 149 of the 165 individuals sampled. Except for the last sampling date, no more than 1 to 2 males were sampled within the roost on each sampling date (Table 4), but males constituted 27% of all individuals on the last collection date (August 29).

**Table 4:** Sample characteristics on each sampling date.

Sampling date (2012)	*M*. *myotis*				*M*. *blythii*				Hybrids			
	F	M	NA	T	F	M	NA	T	F	M	NA	T
**May 22**	38	1	1	**40**	0	0	0	**0**	1	0	0	**1**
**June 20**	24	1	4	**29**	1	0	0	**1**	1	0	0	**1**
**July 10**	35	2	4	**41**	0	0	0	**0**	1	0	1	**2**
**August 03**	32	1	3	**36**	1	0	1	**2**	3	0	0	**3**
**August 29**	6	5	0	**11**	6	2	2	**10**	7	0	0	**7**

F = Female, M = male, NA = not assigned to a gender, T = total number of individuals sampled. Individuals could have been sampled 1 to 4 times across the sampling dates.

The Eggert equation estimated the colony size in accordance with the average visual counting (Table 2), while sequential Bayesian estimations were always higher and surpassed the highest visual count ever made in the roost by the Park officers (530 individuals, May 2009, unpublished data). Eggert estimations showed that from May to August, several hundred bats frequented the roost, while 31 to 43 distinct individuals were sampled, which may represent 10% to 20% of the individuals present at these dates. On the last collection date (August 29), 45 to 47 individuals may have been present within the roost, with 28 individuals sampled.

### Hybrid detection performance

In the simulations, the assignment efficiency of the 11 microsatellite loci by NEWHYBRIDS depended on the criterion and the threshold *Tq* value used (Table 5). The highest *Tq* value (0.90) decreased the assignment efficiency but increased the accuracy. Overall, the efficiency of the 11 microsatellite loci was high, ranging from 0.69 to 1, while accuracy estimates ranged from 0.77 to 1. When no hybrids were assumed in the simulated dataset, NEWHYBRIDS did not detect hybrids (2^nd^ criterion), or it estimated a hybrid proportion close to zero (3^rd^ criterion). When the simulated hybrid proportion was 5% or 10%, the best hybrid proportion estimates were achieved with a 3^rd^ criterion and a threshold of 0.75, with both the efficiency and accuracy > 0.8 and the Type I error ≤ 0.015 (meaning that 0 to 3 individuals were false hybrids). We therefore based the hybrid estimation of the field sample on the use of *Tq* = 0.75 and the 3^rd^ criterion.

**Table 5:** Results of NEWHYBRIDS analyses with simulated samples of N = 200.

Simulated HP (%)	Nb of hybrids in the sample	Criterion	*Tq*	Mean number of hybrids (s.d.)	Estimated HP (%)	Mean squared error	Efficiency		Accuracy		Type I error	Not assigned
							Hybrids	Purebreds	Hybrids	Purebreds		
0	0	2^nd^ criterion	0.75	0.00 (0.00)	0.00	0.00	-	1.000	-	1.000	0.000	0
			0.90	0.00 (0.00)	0.00	0.00	-	0.998	-	1.000	0.000	0
		3^rd^ criterion	0.75	0.10 (0.31)	0.05	0.025	-	1.000	-	1.000	0.001	-
			0.90	0.40 (0.60)	0.20	0.125	-	0.998	-	1.000	0.002	-
5	10	2^nd^ criterion	0.75	7.35 (1.35)	3.68	2.19	0.730	0.995	0.993	0.992	0.000	2
			0.90	6.85 (1.53)	3.43	3.04	0.685	0.984	1.000	0.994	0.005	5
		3^rd^ criterion	0.75	9.40 (1.93)	4.70	0.975	0.845	0.995	0.913	0.992	0.005	-
			0.90	11.80 (3.58)	5.90	3.85	0.875	0.984	0.780	0.994	0.016	-
10	20	2^nd^ criterion	0.75	15.65 (1.63)	7.83	5.36	0.765	0.985	0.978	0.986	0.005	4
			0.90	14.45 (1.8)	7.23	8.46	0.718	0.965	0.993	0.991	0.001	10
		3^rd^ criterion	0.75	20.10 (3.55)	10.05	3.00	0.870	0.985	0.881	0.986	0.015	-
			0.90	24.65 (5.16)	12.33	11.74	0.918	0.965	0.766	0.991	0.035	-

The data are presented as the mean of each measure over 20 repetitions. Abbreviations: HP, hybrid proportions; *Tq*, threshold *q*-value.

### Microsatellite markers and species differentiation

Among the 11 loci used for genotyping analyses, 10 were very polymorphic (Table 6). The allelic richness per locus was the highest in hybrid individuals for 8 of the 11 microsatellite loci (Table 6). The mean allelic richness was 7.66 in *M*. *myotis*, 6.90 in *M*. *blythii* and 8.33 in hybrids. The diversity measured at the 11 microsatellite loci in the three bat types was comparable but not identical (Table 6). Although allele frequencies were estimated on only a small number of *M*. *blythii* and hybrid individuals (see below), locus C113 showed interesting results: allele 97 was quasi-absent from *M*. *myotis* individuals (1/140), with individuals being 100/100 homozygotes for this locus, while allele 97 was predominant for *M*. *blythii* individuals (frequencies of 0.792 for allele 97 and 0.208 for allele 100). Hybrids showed a more balanced frequency distribution, at 0.54 for allele 97 and 0.46 for allele 100. These results correspond with those of Berthier et al. [7], who showed that at locus C113, all of the *M*. *myotis* analysed (N = 80) were homozygotes, while all of the *M*. *blythii* (N = 80) were heterozygous.

**Table 6:** Allelic richness (Ar) and observed (H_O_) and expected (H_E_) heterozygosities measured at 11 microsatellite loci genotyped in a mixed maternity colony of *M*. *myotis* and *M*. *blythii*.

	*M*. *myotis* (N = 140)				*M*. *blythii* (N = 12)				Hybrids (N = 13)			
Locus	Ar	H_O_	H_E_	P	Ar	H_O_	H_E_	P	Ar	H_O_	H_E_	P
**D15**	9.5	0.96	0.89	0.123	6.0	0.42	0.66	0.048	11.5	0.92	0.86	0.997
**F19**	7.2	0.75	0.79	0.025	7.0	0.83	0.82	0.490	9.7	0.77	0.84	0.172
**G2-Mluc**	9.7	0.79	0.87	0.025	8.0	0.92	0.81	0.891	9.8	0.77	0.92	0.047
**G6-Mluc**	6.1	0.74	0.77	0	6.0	0.50	0.71	0.095	6.8	0.38	0.85	0.002
**G31-Mluc**	6.6	0.83	0.81	0.086	6.0	0.83	0.82	0.110	6.0	0.69	0.83	0.086
**B8-Mluc**	10.4	0.76	0.90	0	10.0	0.83	0.89	0.062	9.7	1.00	0.90	0.196
**C113**	1.1	0.01	0.02	0.011	2.0	0.25	0.35	0.261	2.0	0.31	0.53	0.195
**D15-Mluc**	9.3	0.81	0.86	0.137	4.0	0.42	0.59	0.070	8.8	0.85	0.84	0.235
**F19-Mluc**	6.8	0.71	0.77	0.0128	9.0	1.00	0.87	0.563	9.8	0.77	0.84	0.434
**G30-Mluc**	8.7	0.73	0.87	0	7.0	0.92	0.84	0.859	9.8	0.77	0.91	0.003
**H23-Mluc**	8.8	0.86	0.88	0.611	11.0	0.92	0.93	0.771	7.8	0.85	0.85	0.243

The probability (P) of the exact test for HWE is given for each locus and each bat type.

Seven of the 11 loci deviated significantly from HWE in *M*. *myotis* (Table 6): in all of these cases, the heterozygote deficit was significant (P < 0.05), which suggested some level of inbreeding. This result may also be the result of an unbalanced sample comprising only a portion of the bat population (mothers and probably their young on the last sampling date). A heterozygote deficit was also observed for 3 loci in hybrids and 1 locus in *M*. *blythii*. The three types differed significantly in their allele frequencies (G-test, P < 0.001). Of the 165 exact tests performed for genotypic disequilibria at each locus for each bat type, none were significant at the 0.05 level after correction using the FDR procedure.

All but two of the 11 microsatellite markers used in the present study showed a high allele frequency differential (δ ≥ 0.5), which indicated that they possessed good discriminatory power (Table 3). The simulation study provided important insights into the threshold values most appropriate for assigning individuals into three groups (*M*. *myotis*, *M*. *blythii* and hybrids). NEWHYBRIDS clearly distinguished the two parental species as independent genetic units: all of the individuals that were identified as purebreds (*M*. *myotis* and *M*. *blythii* individuals) had a *q*-value ≥ 0.80. The vast majority of *M*. *myotis* individuals (137/140) had a *q*-value ≥ 0.9, with 126/140 having a *q*-value ≥ 0.99. These results were comparable with those for *M*. *blythii*, with 11/12 individuals having a *q*-value ≥ 0.9.

The overall F_ST_ was 0.10, which indicated a moderate genetic differentiation between the three bat types. The F_ST_ was the highest between *M*. *myotis* and *M*. *blythii* (F_ST_ = 0.14) and was lower between hybrids and *M*. *myotis* (F_ST_ = 0.06) and between hybrids and *M*. *blythii* (F_ST_ = 0.03).

### Composition of the mixed maternity colony

**Fig 1:**
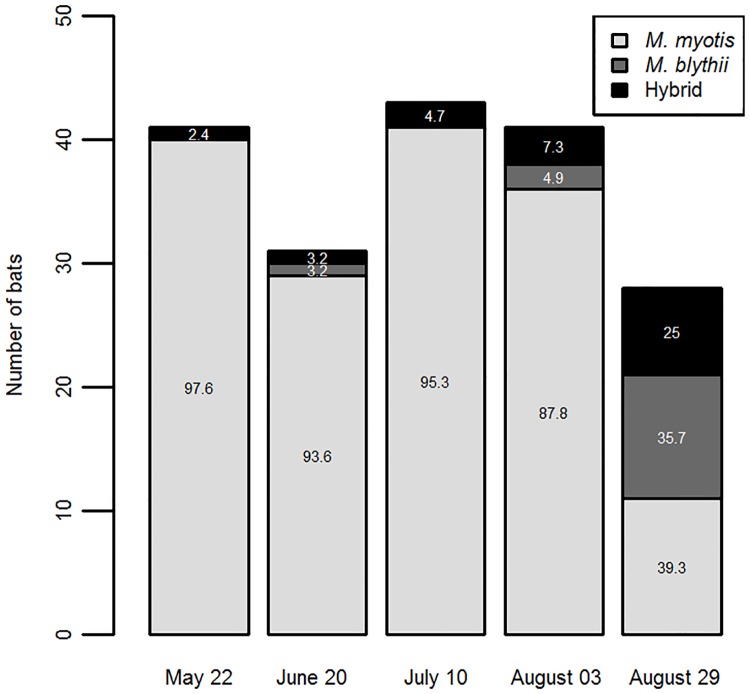
Number of bats sampled on each sampling date and the repartition of *M*. *myotis*, *M*. *blythii* and hybrid individuals. The numbers on each bar represent the proportion (%) of each bat type for a given sampling date.

Considering the three clusters defined using NEWHYBRIDS, the overall frequency of putative hybrid individuals was 7.9% (13/165); *M*. *myotis* represented 84.8% (140/165) of the individuals sampled and 7.3% (12/165) were *M*. *blythii*. The three bat types were unequally distributed across the five sampling dates (Fig 1; Table 4): *M*. *myotis* predominated on the four first sampling dates, while the sample consisted of 39.3% *M*. *myotis*, 35.7% *M*. *blythii* and 25% hybrids at the end of August, when fewer than 50 individuals were present in the roost according to our estimations. The number of hybrid individuals was positively correlated with the number of *M*. *blythii* individuals for a given date (Pearson’s correlation, r = 0.97, df = 3, P = 0.007) but was not significantly related to the number of *M*. *myotis* individuals (r = - 0.84, df = 3, P = 0.073). Molecular sexing found that 6.3% (8/128) of *M*. *myotis* and 22.2% (2/9) of *M*. *blythii* individuals were males. Interestingly, all of the sexed hybrids were females (N = 12).

## Discussion

The present study is the first to focus on a mixed-roosting bat guild to estimate the proportion of hybrid individuals as well as their distribution over time within a maternity roost. Using simulated data, we chose the most appropriate methodology to assign individuals into genetic clusters by minimizing assignment errors. Our results indicate that, although the correct identity of hybrid individuals cannot be guaranteed considering the nature of the samples (*i*.*e*., guano was collected, and individuals were not examined), it is possible to obtain a realistic estimate of the actual proportion of hybrids in our sample using a non-invasive approach. As seen in our simulation studies, in the case of our empirical dataset, NEWHYBRIDS rarely identified false hybrids, causing the estimated proportion of admixed individuals to be very close to the simulated proportion. Based on *M*. *myotis* and *M*. *blythii* genotype clustering, 9 of the 11 microsatellite loci used in the present study had good discriminatory power (δ = 0.50–0.79). They appear to be a good diagnostic kit for the distinction of *M*. *myotis* and *M*. *blythii* when individual capture and handling are difficult to perform. They could also be used in other studies on hybridization between these two bat species together with other markers to distinguish hybrid categories (F1, F2 and backcross).

The overall frequency of hybrid individuals was estimated at 7.9% (13/165) over the study period. This value is consistent with a previous report of 10% of hybrid individuals in two mixed maternity colonies composed of *M*. *myotis* and *M*. *blythii* individuals located in the Swiss Alps and their Italian southern border [7]. This result shows that hybrid individuals may not be rare in natural mixed colonies and calls for the sound confirmation of bat type (*M*. *myotis*, *M*. *blythii* and hybrids) in studies focusing on the ecology of these bat species. In the present study, hybrids showed a low genetic differentiation with the two parental bat species (F_ST_ = 0.03–0.06), whereas the differentiation was moderate but higher between *M*. *myotis* and *M*. *blythii* (F_ST_ = 0.14). An interesting result was observed with locus C113, which was largely monomorphic in *M*. *myotis* but showed polymorphism with unbalanced frequencies in *M*. *blythii* and polymorphism with balanced frequencies in hybrids. This result may suggest more frequent integration of genetic characteristics in *M*. *blythii* than in *M*. *myotis*. These results reiterate those of Berthier et al. [7], who showed that gene introgression might be highly asymmetrical between these two *Myotis* species, with all of the second-generation hybrids being in the direction of *M*. *blythii*, and *M*. *blythii* samples being much more introgressed by *M*. *myotis* genes [7].

The occurrence of hybridization events between *M*. *myotis* and *M*. *blythii* reopens the debate about their taxonomic status. In Europe, both species share mitochondrial lineages as if they were a single species [7, 9, 42]. Berthier et al. [7] supported the idea that the original *M*. *blythii* mtDNA genome has been replaced by local lineages of *M*. *myotis* during their expansion from Asia into Europe, until the original genes were finally lost. However, the authors stressed the maintenance of strong nuclear differentiation between species, which may be explained by the presence of several counter-selected nuclear loci in hybrid individuals, irrespective of their mtDNA background [7]. In the present study, the two species were genetically differentiated with respect to nuclear loci, with a high probability that a purebred individual belongs to each parental cluster. Even if some data suggest that the two *Myotis* species may form a single species, the small number of molecular markers used to measure their genetic differentiation is not sufficient to formally draw conclusions about their taxonomic status. From an evolutionary point of view, the occurrence of hybridization events between *M*. *myotis* and *M*. *blythii* raises questions about how hybrids might be better adapted than either of the parental species to environmental constraints. In this study, the allelic richness was highest in hybrid individuals, which suggested that hybridization could increase the genetic diversity and the chance of carrying alleles favourable to environmental constraints.

Although the life cycle of bat species is not completely understood, it becomes increasingly obvious that hybridization is unlikely to occur in maternity roosts, which essentially host females and offspring. Interspecific matings may be favoured by the mating behaviour of both *Myotis* species. They usually occupy the same colonial roosts in sympatry, either for maternal care or mating [43]. During mating, which occurs in male shelters, males of one species may meet females of the other species [9, 19]. However, the role of maternity colonies in hybridization might not be neutral. In a mixed-species group, the plastic behaviour of young animals facilitates interspecific social interactions, which might be important for the establishment of adult behaviours among species [44]. Future research should therefore investigate social interactions between *M*. *myotis* and *M*. *blythii* in maternity colonies.

Of all the 165 individuals sampled over the five field samplings, the vast majority were *M*. *myotis* (84.8%; 140/165). *Myotis myotis* individuals were predominant from May to the beginning of August (87.8% to 97.6% of the individuals present at a given date) and represented 39.3% of the individuals remaining within the roost at the end of August. Generally, only a few males are present in maternity roosts [45]; therefore, we hypothesize that the high sex ratio observed during this last sampling date for *M*. *myotis* individuals (0.45) reflects the presence of young individuals emancipated from their mother. *Myotis blythii* appeared to be a minority, accounting for 12 of the 165 individuals sampled over the study period. The occurrence of *M*. *blythii* increased over time, with the highest proportion of all of the individuals on the last sampling date. The sex ratio observed for *M*. *blythii* on this date (0.25) may reflect a sampling including both mothers and young. On the last sampling date, the growth of young and the presence of mothers providing maternal care were different between the two species. This result is consistent with current knowledge about the difference in the timing of parturition between the two species: *M*. *blythii* can arrive after *M*. *myotis* and give birth 10 days earlier [13]. Interestingly, all of the sexed hybrid individuals sampled over the study period were female (N = 12). Although the number of hybrids sampled is too small to support a formal conclusion, and female gender is the most likely in maternity roosts, this observation is noticeable with regard to the general distribution of sex ratios in hybrids. Haldane’s rule predicts a lower viability of males, the heterogametic sex, which could lead to a sex ratio that is biased towards females [46]. Extreme sex bias has been reported in the hybrids of two primate species (*Alouatta caraya* and *Alouatta clamitans*; [47]). Further studies on the sex ratio of hybrids are necessary to explore whether such a bias is observable in hybrids of *M*. *myotis* and *M*. *blythii*.

In the present study, we also showed that the timing of roost frequentation and individual abundance was positively correlated between hybrids and *M*. *blythii*. The arrival of both *M*. *blythii* and hybrid individuals in the roost was later than the arrival of *M*. *myotis*. Contrary to genetic and phenotypic characteristics, phenological and ecological traits are unknown in hybrid bats. In other mammalian species, hybrid individuals can adopt behavioural and ecological processes of one of the parental species: hybrids of wildcats and domestic cats present characteristics (size and space use) that are more similar to European wildcats (*Felis silvestris silvestris*) than to domestic cats (*Felis silvestris catus*) [48]. The composition of these hybrids’ diet is intermediate between those of wildcats and domestic cats but is closer to that of one of the parental species, either wildcat [49] or domestic cat [50]. The present study provides an exciting perspective regarding the dynamics of mixed bat colonies and mixed-species groups in general and raises questions about how the behaviour and ecology of hybrids differ from those of the parental species. For example, different social behaviours may be studied, such as the potential association of hybrid individuals with the maternal care provided to the offspring in the roost. As *M*. *myotis* and *M*. *blythii* preferentially prey on different items, hybridization cases offer an interesting opportunity to study how the hybrid diet may differ from that of the parental species and to test a potential expansion of the food niche.

To conclude, this paper illustrates how non-invasive genetic methods can be used to assess demographic parameters (colony size and sex-ratio), as well as population genetics indices and hybridization rate. Although studying hybridization in bats from non-invasive samples presents some limits, like the impossibility to proceed to morphometric measurements on individuals, this sampling method has the advantages of: 1) being easy and fast to enforce, especially when individuals form social groups, 2) allowing the sampling of numerous individuals in a relative short period of time, with no restriction possibly issued from capture (eg. disturbance, ethical, protection, etc.), 3) not needing to coincide bats and researcher presence within the roost, 4) being applicable to all bat species when they form seasonal colonies. In other mammals, occasionally, hair or scat has been used successfully to study hybridization between species difficult to catch [15, 51]. Non-invasive genetic sampling could thus constitute a valuable tool for monitoring hybridization in species of conservation interest.
